# Oxytocin and Social Sensitivity: Gene Polymorphisms in Relation to Depressive Symptoms and Suicidal Ideation

**DOI:** 10.3389/fnhum.2016.00358

**Published:** 2016-07-19

**Authors:** Robyn J. McQuaid, Opal A. McInnis, Kimberly Matheson, Hymie Anisman

**Affiliations:** Department of Neuroscience, Carleton UniversityOttawa, ON, Canada

**Keywords:** depression, suicidal ideation, oxytocin, polymorphism, social connectedness, trauma

## Abstract

Although the neuropeptide oxytocin has been associated with enhanced prosocial behaviors, it has also been linked to aggression and mental health disorders. Thus, it was suggested that oxytocin might act by increasing the salience of social stimuli, irrespective of whether these are positive or negative, thus increasing vulnerability to negative mental health outcomes. The current study (*N* = 243), conducted among white university students, examined the relation of trauma, depressive symptoms including suicidal ideation in relation to a single nucleotide polymorphism (SNP) within the oxytocin receptor gene (OXTR), rs53576, and a SNP on the CD38 gene that controls oxytocin release, rs3796863. Individuals with the polymorphism on both alleles (AA genotype) of the CD38 SNP had previously been linked to elevated plasma oxytocin levels. Consistent with the social sensitivity perspective, however, in the current study, individuals carrying the AA genotype displayed elevated feelings of alienation from parents and peers as well as increased levels of suicidal ideation. Moreover, they tended to report elevated depressive symptoms compared to CC homozygotes. It was also observed that the CD38 genotype moderated the relation between trauma and suicidal ideation scores, such that high levels of trauma were associated with elevated suicidal ideation among all CD38 genotypes, but this relationship was stronger among individuals with the AA genotype. In contrast, there was no relationship between the OXTR SNP, rs53576, depression or suicidal ideation. These findings support a social sensitivity hypothesis of oxytocin, wherein the AA genotype of the CD38 SNP, which has been considered the “protective allele” was associated with increased sensitivity and susceptibility to disturbed social relations and suicidal ideation.

## Introduction

Oxytocin, a neuropeptide that has widespread central and peripheral effects, has frequently been examined in relation to prosocial behaviors. Individuals who receive intranasal oxytocin administration display enhanced generosity (Zak et al., 2007), trust (Kosfeld et al., 2005), empathy (Domes et al., 2007; Shamay-Tsoory et al., 2013), and helping behavior (Riem et al., 2013). However, oxytocin does not consistently enhance prosociality, having been shown to increase envy and gloating (Shamay-Tsoory et al., 2009), lying to benefit one’s in-group (Shalvi and De Dreu, 2014), defensive aggressive behaviors towards competing outgroup members (De Dreu et al., 2010), and aggression and violent behaviors towards a romantic partner among individuals high in trait aggression (DeWall et al., 2014). To explain the divergent outcomes associated with oxytocin, it was suggested that aside from any prosocial features of this hormone, oxytocin increases sensitivity to social cues, such that both positive and negative events will become more salient and have more dramatic consequences (Averbeck, 2010; Bartz et al., 2011a; Cardoso et al., 2014; McQuaid et al., 2014).

The social sensitivity perspective associated with oxytocin has also been apparent in analyses involving a genetic variant of the oxytocin receptor gene (OXTR). In this regard, while certain oxytocin genotypes might be considered as “vulnerable” in a negative environment, (Caspi et al., 2010), these genotypes might also be more sensitive to, and thus, influenced by a positive environment. This perspective is in-line with the biological sensitivity theory (Boyce and Ellis, 2005) and a plasticity theory (Belsky and Pluess, 2009). In this regard, a single nucleotide polymorphism (SNP) within the OXTR, rs53576, which involves a guanine (G) to adenine (A) substitution, was associated with several prosocial behaviors. In particular, individuals with two copies of the G allele of this OXTR SNP displayed beneficial attributes, including high levels of trust (Krueger et al., 2012), empathy (Rodrigues et al., 2009; Smith et al., 2014), self-esteem, and lower negative affect (Saphire-Bernstein et al., 2011). However, individuals with the GG genotype who experienced severe childhood maltreatment displayed *greater* disorganized attachments and *increased* emotional dysregulation compared to A carriers (Bradley et al., 2011). Similarly, G carriers who experienced early-life maltreatment reported higher depressive scores compared to individuals with the AA genotype (McQuaid et al., 2013), as well as increased reactivity to social ostracism (McQuaid et al., 2015). These findings, like the effects resulting from oxytocin administration, suggest that G carriers may be more prosocial in certain environments, but in the face of negative experiences, the more socially sensitive G allele carriers may also be more vulnerable to adverse outcomes.

In addition to the OXTR gene, the CD38 gene has been implicated in relation to oxytocin neural transmission (Jin et al., 2007). This gene is required for oxytocin secretion, as CD38 knockout mice display greatly reduced cerebrospinal fluid (CSF) and plasma oxytocin, and these mice also exhibited deficits in social memory and recognition (Jin et al., 2007). Consistent with these findings, in humans peripheral CD38 gene expression was positively correlated with oxytocin levels (Kiss et al., 2011), and a common variant of a SNP involving a cytosine (C) to adenine (A) switch on the CD38 gene, rs3796863, was related to autism spectrum disorder (ASD), which involves deficits in social processing (Lerer et al., 2010; Munesue et al., 2010). Based on this finding, the C-allele, although more common, has been determined to be the “risk” allele and was associated with lower CD38 expression in lymphoblastoid cells obtained from individuals with ASD (Lerer et al., 2010). Although the literature examining the CD38 rs3796863 SNP is limited, and has largely been assessed in relation to ASD, the C allele homozygotes were found to display reduced plasma oxytocin levels and less sensitive parenting techniques (Feldman et al., 2012). It was revealed, however, that upon experiencing chronic stress, A carriers of the CD38 SNP displayed elevated social anxiety compared to CC homozygotes, which was consistent with a social sensitivity perspective (Tabak et al., 2016).

The association of oxytocin with disorders comprising disturbed social functioning is not limited to autism (Guastella et al., 2010; Preti et al., 2014), but was also apparent in relation to schizophrenia (Feifel et al., 2012), depressive disorders (Scantamburlo et al., 2007; McQuaid et al., 2014), and suicidal behaviors (Lee et al., 2009; Jokinen et al., 2012). In the latter regard, individuals who had previously attempted suicide displayed lower CSF oxytocin concentrations compared to healthy controls (HCs; Lee et al., 2009). Furthermore, low CSF oxytocin concentrations were associated with suicide intent among males who had previously attempted suicide, although there were no differences in CSF or plasma oxytocin concentrations between individuals who had completed suicide and those who attempted but survived (Jokinen et al., 2012).

Given the relationship between oxytocin and both depression and suicidality, in the current investigation it was hypothesized that genetic variants of the OXTR SNP rs53576 and the CD38 gene SNP rs3796863 would be related to depression and suicidal ideation among young adults. Furthermore, as traumatic events may be a risk factor for suicidal behaviors (Dube et al., 2001; Seedat et al., 2005) and individuals who have experienced trauma displayed lower CSF oxytocin concentrations (Heim et al., 2008), we hypothesized that the relation between traumatic life events and depression suicidal ideation would be moderated by the OXTR and CD38 genotypes. As oxytocin has been implicated in social bonding and social support, we also explored whether the OXTR and CD38 genotypes were associated with altered relationships to parents and peers through feelings of alienation. These relations were examined among University students enrolled in a first year psychology course, as the transition to university is a particularly stressful period (Compas et al., 1986) associated with high levels of distress (Kidder et al., 2000).

## Materials and Methods

### Participants

Due to population stratification effects in relation to genotypes, the current study examined a homogeneous White sample comprising 154 females and 89 males, with a mean age of 19.17 (SD = 2.04). Participants were Carleton University first year students who were recruited through the university’s online computerized system.

### Procedure

Upon signing informed consent, participants completed a series of demographic questions, including a question regarding whether they currently had a psychological disorder, as well as measures of current depressive symptoms including suicidal ideation, traumatic life events and feelings of alienation from parents and peers. Following completion of the questionnaires, saliva samples were obtained for later genotyping. Participants were then debriefed and compensated with course credit. The current study was approved by the Carleton University Ethics Committee for Psychological Research.

### Genotyping

Saliva samples for genotyping were collected using Norgen collection kits (Norgen Biotek Corp., Thorold, ON Canada). Genomic DNA was extracted from the sample collection kit according to the manufacturer’s instructions and diluted to approximately equal concentration (10 ng/μL), and genotyping was performed at the McGill University and Génome Québec Innovation Centre (Montreal, QC, Canada). Using polymerase chain reaction (PCR) the DNA was amplified, and QIAXcel was used to determine amplification status. Shrimp alkaline phosphatase was used to remove all unincorporated dNTPs. One probe per marker was used to do a single base extension and the product was desalted using 6 mg of resin. The product was spotted on a Sequenom 384-well chip using a Samsung Nanodispenser and the chip read by a Mass Spectrometer. A manual analysis was done for each marker. Primer sequences were as follows: CD38 forward: ACGTTGGATGGTTGCTGCTCCTGCTGTTTT, CD38 reverse: ACGTTGGATGAAGGTGCACAGACCACTTAG, CD38 probe: TCCTGCTGTTTTTTTGACCA, OXTR forward: ACGTTGGATGTCCCCATCTGTAGAATGAGC, OXTR reverse: ACGTTGGATGGCACAGCATTCATGGAAAGG, OXTR probe: CTCTGTGGGACTGAGGA.

The allele distribution of the CD38 rs3796863 SNP was 106 CC individuals (33 males, 73 females) and 105 CA individuals (50 males, 55 females) and 26 AA individuals (4 males, 22 females). These genotype distributions met Hardy-Weinberg Equilibrium expectations, $\chi_{\left(1\right)}^{2}$ = 0, *p* > 0.05. Six individuals were excluded from analyses that included genotype as an independent variable since the genotype could not be determined from these particular samples. For the OXTR SNP, rs53576, the genotype distributions were; 109 GG individuals (35 males, 74 females), 106 AG individuals (43 males, 63 females), and 26 AA individuals (10 males, 16 females). Two individuals could not be genotyped for this OXTR SNP and thus were excluded from any analyses including genotype. These genotype distributions also met Hardy-Weinberg Equilibrium expectations, $\chi_{\left(1\right)}^{2}$ = 0, *p* > 0.05. The presence of the two SNPs were independent of one another, $\chi_{\left(1\right)}^{2}$ = 2.88, *p* =* 0*.59.

### Measures

#### Depressive Symptoms

The 21-item Beck depression inventory (BDI; Beck et al., 1961) was used to assess depressive symptoms. For each item participants responded to one of four options which ranged from low to high depression symptomatology. Total scores were calculated by summing across all items (*α* = 0.90). To be sure that findings pertaining to depressive symptoms were not influenced by feelings of suicidal ideation, analyses were also carried out excluding the suicidal ideation question from the total BDI score, and both types of analyses yielded the same results. Suicidal ideation was determined through the use of a question on the 21-item BDI that assesses suicidal thoughts and behaviors. This question includes the following response options: 0 (I don’t have thoughts of harming myself), 1 (I have thoughts of harming myself but I would not carry them out), 2a (I feel I would be better off dead), 2b (I have definite plans about committing suicide), 2c (I feel my family would be better off if I were dead), and 3 (I would kill myself if I could).

#### Number of Different Types of Traumas Experienced

The Traumatic Life Events Questionnaire (TLEQ; Kubany et al., 2000) was used to measure the number of different types of traumatic events experienced. This measure includes 23 types of traumatic events experienced (e.g., natural disasters, assaults, death of a loved one). Responses for each item can vary from 0 (never having experienced the event), through to 6 (having experienced the event more than 5 times). Items were summed to create a frequency score for traumatic life events.

#### Alienation from Parents and Peers

A 15 item subscale from the Inventory of Parent and Peer Attachment (IPPA; Armsden and Greenberg, 1987) was used to assess the quality of attachment to parents and peers in one domain, namely alienation. A mean score was determined for parental (*α* = 0.88) and peer (*α* = 0.81) alienation.

## Statistical Analyses

Statistical analyses were performed using SPSS for Windows 18.0 (SPSS Science, Chicago, IL, USA). Analyses assessing genotype differences on parental and peer alienation, depression scores and suicidal ideation were performed using a one-way analysis of variance (ANOVA), followed by Bonferroni corrected *t*-tests for any significant outcomes. Correlational analysis was performed using Pearson product moment correlations. Hierarchical linear regressions were used to analyze moderation effects, and the significant moderations were followed up using a web utility for simple slopes (Preacher et al., 2006). Although it is ideal to keep all three genotypes separate whenever possible (for a discussion on this topic, see McQuaid et al., 2015), for moderation analyses, which require a dichotomous moderator, genotypes were collapsed into two groups. For the CD38 polymorphism, C carriers were pooled together and compared to individuals with the AA genotype, as this approach was the best fit for the data. Specifically, individuals with the AA genotype differed on a number of variables of interest compared to both individuals with the CC or AC genotypes. Further, for the OXTR polymorphism the G carriers were pooled and compared to those with the AA genotype, which also appeared to fit the data better. Moderation analyses were also conducted collapsing according to the dominant genotype for both SNPs and the results remained consistent.

## Results

As presented in Table 1, scores on the 21-item BDI were correlated to trauma scores and the item assessing suicidal ideation from the BDI, as well as feelings of alienation from both parents and peers.

**Table 1:** **Pearson correlations among traumatic life events, parental and peer alienation, suicidal ideation and depressive symptoms**.

	1	2	3	4	5
1. Traumatic life events	—				
2. Parental alienation	0.33**	—			
3. Peer alienation	0.21**	0.68**	—		
4. Suicidal ideation	0.35**	0.53**	0.42**	—	
5. Depressive symptoms	0.35**	0.67**	0.64**	0.67**	—

**p < 0.001.

### CD38 SNP rs3796863

**Figure 1:**
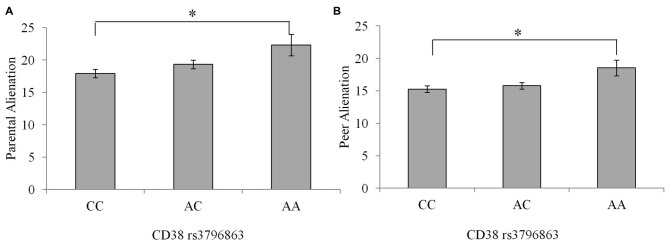
**Alienation from parents (A) and peers (B) among individuals with the CC, AC, or AA CD38 genotypes**. Data represent means ± SEM. *p = 0.01 compared to individuals with the CC genotype.

There were no differences between genotype groups regarding the incidence of a current psychological disorder $\chi_{\left(2\right)}^{2}$ = 1.46, *p* = 0.48. As well, there were no Sex × CD38/OXTR genotype interactions in relation to peer or parental alienation, suicidal ideation or depression scores. Differences existed between the CD38 genotypes on feelings of alienation from parents and peers, *F*_(2,234)_ = 4.30, 4.12, *p’s* = 0.02, *η*^2^ = 0.04 and 0.03, respectively. In particular, as shown in Figure 1, compared to individuals homozygous for the C allele, those with the AA genotype reported greater feelings of alienation from parents and peers *p’s* = 0.01. Individuals with the AC genotype did not differ in levels of alienation from the other genotypes.

**Figure 2:**
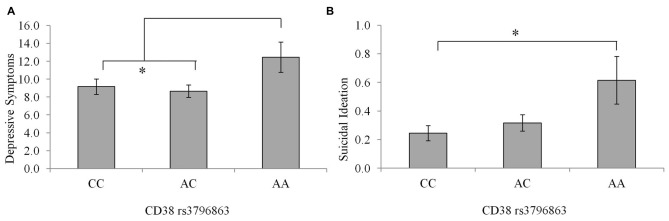
**Depressive symptoms (A) and suicidal ideation scores (B) among individuals with the CC, AC, or AA CD38 genotypes**. Data represent means ± SEM. *p < 0.05 compared to CC homozygotes.

Depression scores among individuals with the CC and AC genotype were very similar, as seen in Figure 2, and tended to be lower relative to that of the AA genotype, although these differences did not reach an acceptable level of significance, *F*_(2,234)_ = 2.32, *p* = 0.10. This said, when individuals carrying the AA genotype were compared to the pooled AC and CC genotypes, the levels of depression (*M* = 12.46; SE = 1.67 and *M* = 8.90 SE = 0.55, respectively) were significantly higher in the AA individuals, *F*_(1,235)_ = 4.46, *p* = 0.03, *η*^2^ = 0.02. Suicidal ideation scores differed between CD38 genotypes, although the amount of variance accounted for was small, *F*_(2,233)_ = 3.95, *p* = 0.02, *η*^2^ = 0.03. As displayed in Figure 2, individuals with the CC genotype displayed lower suicidal ideation scores compared to AA individuals (*p* = 0.02), while ideation did not differ significantly between the AC and AA individuals (*p* = 0.07).

**Figure 3:**
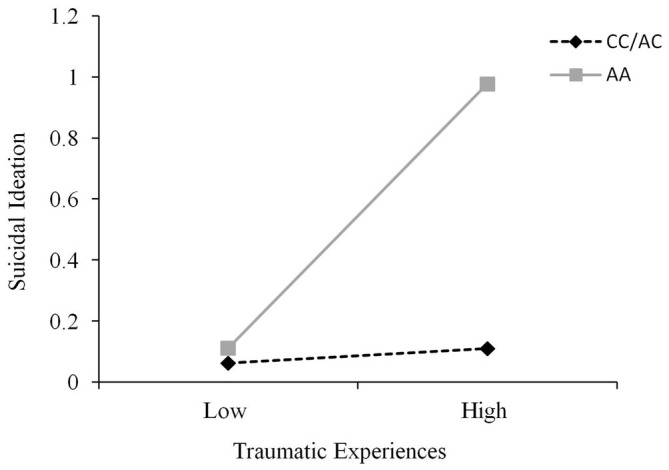
**The relation between traumatic life events and suicidal ideation scores as a function of CD38 rs3796863 genotype (CC/AC vs. AA)**. The simple slopes analyses revealed that the relation between trauma and suicidal ideation was significant among all genotypes but this effect was stronger among those with the AA genotype.

A fairly wide range of traumas were recorded using the TLEQ, and it was not unusual for participants to experience a given event on more than a single occasion. These occurrences were scored as reflecting multiple trauma encounters. Approximately 15% of individuals reported a score of 1 or less severe events, whereas 60% reported such experiences on 2–9 occasions, and approximately 25% reported a score of greater than 10 events. As expected, the number of these events did not differ across genotypes, *F*_(2,232)_ = 0.20, *p* = 0.82. To examine the possible moderating effect of the CD38 genotype on the relation between trauma and both depression and suicidal ideation, hierarchical linear regressions were conducted where CD38 genotype and trauma scores were entered on the first step and the CD38 Genotype × Trauma interaction term was entered on the second step. It was found that trauma did not interact with CD38 genotype to predict depression scores, Δ*R*^2^ = 0.002, Δ*F*_(1,231)_ = 0.44, *p* = 0.51. However, the CD38 genotype moderated the relationship between trauma and suicidal ideation, Δ*R*^2^ = 0.03, *b* = 0.41, *t* = 2.85, *p* < 0.01. Follow-up simple slopes analyses, as shown in Figure 3, revealed that the relation between low levels of trauma and suicidal ideation was similar among all genotypes. Furthermore, although suicidal ideation scores at high levels of trauma were significantly elevated among all genotypes, this relation was stronger among individuals with the AA genotype (*b* = 0.07, 95% confidence interval (CI): 0.04, 0.11) than that found among C carriers (*b* = 0.02, 95% CI: 0.01, 0.03). This analysis was also conducted controlling for depression scores as a covariate and the CD38 genotype × Trauma interaction in predicting suicidal ideation remained significant, *p* < 0.01. In effect, the relation between CD38 genotype and trauma in predicting suicidal ideation occurred above and beyond the influence of depression.

### OXTR SNP rs53576

There were no genotype differences evident in relation to currently having a psychological disorder, $\chi_{\left(2\right)}^{2}$ = 0.66, *p* = 0.72, or in traumatic life event scores, *F*_(2,236)_ = 0.17, *p* = 0.85. Unlike the relation observed for the CD38 gene, feelings of alienation to parents and peers were similar among OXTR genotype groups, *F*_(2,238)_ = 0.03, *p* = 0.97, and *F*_(2,238)_ = 0.56, *p* = 0.57, respectively. Depression scores were very similar for the GG (*M* = 9.25; *SE* = 0.78), AG (*M* = 9.13; *SE* = 0.79), and AA (*M* = 9.92; *SE* = 1.60) OXTR genotypes, *F*_(2,238)_ = 0.09, *p* = 0.91. Likewise, suicidal ideation scores did not differ across OXTR genotypes, *F*_(2,237)_ = 0.006, *p* = 0.99, GG (*M* = 0.32; *SE* = 0.56), AG (*M* = 0.32; *SE* = 0.66), and AA (*M* = 0.31; *SE* = 0.62).

As in the case of the CD38 SNP, it was of interest to examine whether an OXTR genotype × Trauma interaction would predict depression and suicidal ideation scores. Once more, trauma did not interact with OXTR genotype to predict depression scores, Δ*R*^2^ = 0.00, Δ*F*_(1,235)_ = 0.09, *p* = 0.76. Similarly, there was no interaction between OXTR genotype and trauma to predict suicidal ideation, Δ*R*^2^ = 0.002, Δ*F*_(1,234)_ = 0.57, *p* = 0.45. This analysis was repeated controlling for depression scores as a covariate, and the results remained identical.

## Discussion

Oxytocin disturbances have been linked to depression (McQuaid et al., 2014) and to suicidal intent (Jokinen et al., 2012). As oxytocin has been linked to prosociality there were indeed several reports suggesting that oxytocin levels ought to be reduced in association with depression, and that intranasal oxytocin treatment might function in an antidepressant capacity or as an adjunct to other treatments (Baskerville and Douglas, 2010; Scantamburlo et al., 2011). This said, the view was also expressed that oxytocin might serve to increase the salience of environmental stimuli or the response to such signals to promote context dependent mood changes (Averbeck, 2010; Bartz et al., 2011a; Cardoso et al., 2014).

It was observed in the current study that individuals with the AA genotype for the CD38 SNP reported greater depression and suicidal ideation compared to C carriers. Further, the link to suicidal ideation was especially notable when high levels of trauma had been experienced by those with the AA genotype. At first blush this finding might seem counterintuitive, as A carriers of this particular SNP were reported to exhibit greater oxytocin levels (Feldman et al., 2012), which were viewed as being protective against social disturbances, including ASD (Lerer et al., 2010; Munesue et al., 2010). However, individuals with the AC or AA genotype for the CD38 SNP were also found to exhibit more sensitive parenting methods (Feldman et al., 2012), and displayed faster reaction times to emotionally relevant social stimuli (Sauer et al., 2012). These findings support the notion that the A carriers of the CD38 SNP may be more sensitive to social stimuli, whereas the less socially reactive CC homozygotes, who may have lower plasma oxytocin levels, are at risk for ASD. The view linking social sensitivity and psychopathology was supported by the finding that in the context of chronic stress, the A carriers of CD38 SNP displayed elevated social anxiety (Tabak et al., 2016). The current finding that individuals with the AA genotype reported greater depression scores as well as elevated suicidal ideation that was still more pronounced in the context of high levels of trauma, is likewise consistent with a social sensitivity perspective concerning the actions of oxytocin. In line with this view, genetic factors might influence plasticity to environmental stimuli, and “for better or for worse” affect mood outcomes (Belsky and Pluess, 2009).

In addition to somewhat elevated levels of depression and increased suicidal ideation, individuals with the AA genotype for the CD38 SNP also reported greater feelings of alienation from both parents and peers. This is particularly relevant as low parental expressive support has been related to elevated suicidal ideation (Winfree and Jiang, 2010) and low levels of social connectedness and support have been associated with more frequent suicide attempts (Compton et al., 2005). Indeed, having strong social identities and ties to social networks was accompanied by reduced depressive symptoms (Cruwys et al., 2013), and thus it might be expected that perceptions of disturbed parental and peer relationships among individuals with the AA genotype would also be related to depression and suicidal ideation. Although speculative, this may also be indicative of the AA carriers being more sensitive, and therefore perceiving (or misperceiving) greater levels of alienation. In essence, given the involvement of oxytocin in social bonding and attachment (Insel and Hulihan, 1995; Carter, 1998), it is possible that the link between oxytocin and mental health disturbances occur through altered social functioning (McQuaid et al., 2014).

While not dismissing the view that oxytocin promotes prosocial behaviors, the effects of oxytocin depend on contextual and person factors (Bartz et al., 2011a), and may elicit antisocial behaviors among certain individuals (Bartz et al., 2011b). Indeed, it seems that oxytocin could instigate adverse outcomes among individuals who do not display social processing deficits, possibly because oxytocin treatment renders them inappropriately sensitive within social situations (Cardoso et al., 2014). Moreover, although intranasal oxytocin treatment may elicit prosocial behaviors in humans and in animals (MacDonald and MacDonald, 2010; Kent et al., 2016), it was also reported that in prairie voles intranasal oxytocin promoted maternal aggression in response to an intruder (Jia et al., 2008), and among mice with increased oxytocin receptors elevated fear and anxiety was observed in response to a social stressor (Guzmán et al., 2013).

In contrast to the CD38 gene, in the current investigation, individuals carrying the G allele for the OXTR SNP, rs53576, did not display elevated depressive symptoms including suicidal ideation scores. Although this is consistent with our previous findings (McQuaid et al., 2013; McInnis et al., 2015), it contrasts with a prior report that A-allele carriers displayed greater depressive symptomatology (Saphire-Bernstein et al., 2011). These variations may be due to the different measures used to assess depressive symptoms. Furthermore, in the context of high levels of trauma, the G carriers, who are often thought to be more socially sensitive, did not display elevated suicidal ideation or depression scores. Although it was not predicted that this SNP would be related to suicidal ideation, given our earlier finding that G carriers displayed elevated depressive symptoms upon experiencing early-life maltreatment (McQuaid et al., 2013), we had expected that G carriers who had experienced high levels of trauma, would display elevated depression scores. In this regard, it had been reported that African American GG homozygotes of this OXTR SNP who experienced severe childhood maltreatment displayed elevated emotional dysregulation and disorganized attachment (Bradley et al., 2011). The current investigation assessed traumatic life events that comprised non-interpersonal events (e.g., such as a natural disaster), as well as interpersonal events, such as the death of a loved one. However, the form trauma assessed differed appreciably from our earlier study in which we assessed the relation of the OXTR SNP to childhood maltreatment perpetrated by a parental figure or guardian. Indeed, in our earlier report childhood maltreatment was accompanied by feelings of distrust among the sensitive G carriers, which mediated the relation between maltreatment and depressive symptomatology (McQuaid et al., 2013). In the current study, we also assessed whether the OXTR SNP was related to depression scores and trauma exclusively comprising interpersonal events. Once again, the findings were non-significant, likely suggesting that it is not only the interpersonal nature of a trauma, but also the source of maltreatment (i.e., parent or guardian) that was particularly pertinent among G carriers of the OXTR SNP.

Interestingly, the relationship between the AA genotype of the CD38 SNP and experiences of trauma on suicidal ideation was not recapitulated in relation to depression. Although suicidal ideation is strongly linked to depression, it is also associated with other psychiatric disorders as well as non-psychiatric conditions (e.g., in association with chronic illness), and not all instances of depression are accompanied by suicidal ideation (Mann, 2003). Diverse neurobiological mechanisms might similarly be linked to suicidal ideation, varying with the context in which this occurs (Dwivedi, 2012). For example, biological correlates of suicide have included altered serotonin and GABA receptor variations (Anisman et al., 2012) as well as that of the neurotrophin FGF-2 (Evans et al., 2004). In addition, suicidal ideation has been associated with SNPs on the serotonin transporter and receptor genes (Wang et al., 2009; Kim et al., 2014), in addition to methylation of a gene coding for brain derived neurotrophic factor (BDNF; Kim et al., 2015).

There are several limitations concerning the conclusions that could be drawn from the present findings. The functionality of the OXTR and the CD38 SNP are not fully understood. It has been suggested that the OXTR SNP might be involved in transcriptional suppression (Mizumoto et al., 1997), but it is also possible that the effects are associated with linkage(s) to other functional OXTR SNPs (Lin et al., 2007). Furthermore, although elevated plasma oxytocin occurs in A carriers of the CD38 SNP (Feldman et al., 2012), this finding bears replication, and this needs to conducted in samples in which oxytocin is extracted from plasma before the assay is conducted. Indeed, unextracted plasma oxytocin levels can be more than 100-fold higher compared to the oxytocin present in extracted sample (Szeto et al., 2011; McCullough et al., 2013), likely owing to molecules other than oxytocin also being detected. A second limitation of the present study concerned the moderate sample size used. It certainly would have been advantageous to have a larger number of minor allele carriers for the CD38 and OXTR SNPs. Moreover, a larger sample would have allowed for a more detailed analysis of sex differences that might have existed concerning relationships between the polymorphisms, depression and suicidal ideation. Further, even though suicidal ideation in those with the AA genotype of the CD38 SNP was exacerbated in the context of high levels of trauma, this does not necessarily translate into elevated intent, nor is it necessarily linked to later suicidal efforts. Moreover, suicidal ideation was measured based on a single item from the BDI scale. Ideally, multiple items and/or a scale focused on suicidal ideation would have been used. Finally, in the present investigation we adopted the TLEQ to measure traumatic events, although it could be questioned whether all the items actually reflected trauma outcomes, such as those leading to posttraumatic stress disorder, and might instead have reflected strong stressors.

These caveats notwithstanding, the present findings are consistent with the view that although oxytocin may well have prosocial actions, it might also function to increase the salience of social cues, or dispose individuals to greater sensitivity or reactance to environmental events. Thus, oxytocin could act against or favor well-being depending upon social and contextual factors. For instance, oxytocin could be associated with feelings of alienation becoming more apparent, thereby increasing susceptibility to negative mental health outcomes.

## Author Contributions

RJM, OAM and HA contributed to the inception and design of the current experiment. Testing and data collection were performed by RJM and OAM. Data analysis and the writing of the manuscript were performed by RJM, OAM, KM, and HA. All authors approved the final version of the article for submission.

## Funding

This research was supported by the Canadian Institutes of Health Research (CIHR) and the Natural Sciences and Engineering Research Council of Canada (NSERC). The funding sources had no further role in the study or in the writing of this article.

## Conflict of Interest Statement

The authors declare that the research was conducted in the absence of any commercial or financial relationships that could be construed as a potential conflict of interest.
